# Protocol for a systematic review on the effect of demand generation interventions on uptake and use of modern contraceptives in LMIC

**DOI:** 10.1186/s13643-015-0102-7

**Published:** 2015-09-28

**Authors:** Loubna Belaid, Alexandre Dumont, Nils Chaillet, Vincent De Brouwere, Amel Zertal, Sennen Hounton, Valéry Ridde

**Affiliations:** Maternal and Reproductive Health Unit, Public Health Department, Institute of Tropical Medicine, 155 Nationalestraat, 2000 Antwerp, Belgium; UMR 216 IRD–Université Paris Descartes, 4 Avenue de l’Observatoire, 75 006 Paris, France; Département d’obstétrique et gynécologie et département de Médecine de famille et médecine d’urgence, Faculté de médecine et des sciences de la santé, Centre de recherche du CHUS: Axe Santé: populations, organisation, pratiques, Université de Sherbrooke, Sherbrooke, Canada; Centre de recherche du CHUM, Axe Évaluation, Systèmes de soins et services, Université de Montréal, 850, rue Saint Denis-Tour S, Local S03-814, Montréal, QC H2X 0A9 Canada; Commodity Security Branch, Technical Division, United Nations Population Fund, 605 3rd Avenue, New York, NY 10158 USA; Institut de recherche en santé publique de l’Université de Montréal (IRSPUM), 7101 Avenue du Parc, bureau 3187-03, Montréal, Québec H3N1X9 Canada; ESPUM (école de santé publique de l’Université de Montréal), Montréal, Canada

**Keywords:** Family planning, Demand generation intervention, Implementation, Cost-effectiveness, Impact, Contraception use, Low- and middle-income countries, Systematic review, Protocol

## Abstract

**Background:**

Despite a global increase in contraception use, its prevalence remains low in low- and middle-income countries. One strategy to improve uptake and use of contraception, as an essential complement to policies and supply-side interventions, is demand generation. Demand generation interventions have reportedly produced positive effects on uptake and use of family planning services, but the evidence base remains poorly documented. To reduce this knowledge gap, we will conduct a systematic review on the impact of demand generation interventions on the use of modern contraception. The objectives of the review will be as follows: (1) to synthesize evidence on the impacts and costs of family planning demand generation interventions and on their effectiveness in improving modern contraceptive use and (2) to identify the indicators used to assess effectiveness, cost-effectiveness, and impacts of demand generation interventions.

**Methods/design:**

We will systematically review the public health and health promotion literature in several databases (e.g., CINAHL, Medline, EMBASE) as well as gray literature. We will select articles from 1970 to 2015, in French and in English. The review will include studies that assess the impact of family planning programs or interventions on changes in contraception use. The studied interventions will be those with a demand generation component, even if a supply component is implemented. Two members of the team will independently search, screen, extract data, and assess the quality of the studies selected. Different tools will be used to assess the quality of the studies depending on the study design. If appropriate, a meta-analysis will be conducted. The analysis will involve comparing odd ratios (OR)

**Discussion:**

The systematic review results will be disseminated to United Nations Population Fund program countries and will contribute to the development of a guidance document and programmatic tools for planning, implementing, and evaluating demand generation interventions in family planning. Improving the effectiveness of family planning programs is critical for empowering women and adolescent girls, improving human capital, reducing dependency ratios, reducing maternal and child mortality, and achieving demographic dividends in low- and middle-income countries.

**Systematic review registration:**

This protocol is registered in PROSPERO (CRD 42015017549).

## Background

Despite a global increase of approximately 11 % in the use of modern contraception from 1994 to 2012, its prevalence remains low in many low-income and some middle-income countries (LMIC), and especially in sub-Saharan African countries such as Mali (9.3 %), Chad (5.5 %), Sierra Leone (7.6 %), and South Sudan (4.9 %), where its prevalence is under 10 %. Although progress has been made in Western Asia, Africa, and Latin America (including the Caribbean), where contraceptive prevalence rates were estimated in 2010 at 57.6, 30.9, and 73.2 %, respectively, the unmet need for contraception is still significant [1], affecting 34.2 % of women in Western Asia, 30 % in Africa, and 10.4 % in Latin America [2].

Yet an increase in contraception use could prevent up to one third of maternal deaths [3]. A study assessing the impact of contraceptive use on maternal mortality in 172 countries reported that 342,203 women died of maternal causes in 2008. Using modeling, that study estimated that contraception use had prevented 272,040 (uncertainty interval 127,937–407,134) maternal deaths, and that meeting unmet contraception need could have prevented a further 104,000 maternal deaths per year (29 % reduction) [4]. Family planning acts positively on maternal mortality by reducing the probability of pregnancy and thereby avoiding its complications, by reducing the risk of unsafe abortions, and by delaying first pregnancies, especially among adolescents [5].

Family planning programs combine enabling environments (policies, strategies, etc.) with both supply and demand interventions [5]. The objective of supply-side activities is to ensure the availability, accessibility, and quality of contraceptive methods for the population. These activities are often described in terms of supply-chain management systems, access, quality, and costs [5]. Some examples of supply-side interventions are as follows: developing cost-effective interventions for integrating family planning with maternal and newborn health, ensuring the availability of family planning products and organizing their community-based distribution, and improving health providers’ technical skills [6].

Demand generation interventions are generally classified into three categories: interpersonal communications, mass media, and innovative financing approaches [5, 7]. Interpersonal communications include group discussions, one-on-one discussions, small group sessions, facilitator-led curricula, and health worker counselling. The aim of this category of interventions is to change people’s attitudes toward family planning. In one study of an intervention in Nigeria consisting mainly of interpersonal communication (entertainment education), an increase in modern contraceptive use was reported in each of the intervention sites, ranging from 2.3 to 15.5 %, 3 years after implementation [8].

Mass media interventions are aimed at changing people’s perceptions and attitudes toward family planning and increasing their knowledge about sexual and reproductive health. For instance, a multi-country study (Nigeria, India, Kenya, Senegal) reported an increase in modern contraceptive use after implementation of a television program conveying family planning messages (OR = 1.24; *p* < 0.05) [6].

Innovative financing interventions to generate demand include vouchers, cash transfers, social transfers, and micro-credit designed to improve access to and use of modern contraception methods [5]. A study in Pakistan reported a 28.4 % increase in modern contraception use and an overall contraceptive rate of 19.6 % after implementation of a voucher scheme; the intervention included social franchising (training private providers and social marketing). The sample in that study, conducted in four districts in Punjab and Sindh provinces, consisted of 4992 women of reproductive age [9].

Demand generation interventions have had positive effects on indicators related to sexual and reproductive health in terms of use of reproductive health services (e.g., family planning) and increased knowledge about human immunodeficiency virus (HIV) and sexually transmitted infections (STIs) [10, 11]. However, the heterogeneity in the designs of studies assessing demand-side interventions and the lack of evidence on indicators used to measure the outcomes of such interventions make it difficult to draw overall conclusions about their effectiveness [5, 11].

As part of its family planning strategy, the United Nations Population Fund (UNFPA) has commissioned this systematic review on the effectiveness, cost-effectiveness, and impact of family planning demand generation interventions in LMICs. The results of the systematic review will be disseminated to UNFPA program countries and will contribute to the development of a guidance document and programmatic tools for planning, implementing, and evaluating demand generation interventions in family planning.

## Methods/design

### Objectives and research questions

The objectives of the systematic review will be as follows: (1) to synthesize evidence on the impacts and costs of family planning demand generation interventions and on their effectiveness in improving access and uptake of modern contraception use; and (2) to identify the indicators used to assess effectiveness, cost-effectiveness, and impacts of demand generation interventions.

The research questions are as follows: what are the impacts of family planning demand generation interventions on indicators of contraceptive use? What are the direct and indirect costs of such interventions?

### Design

**Fig. 1:**
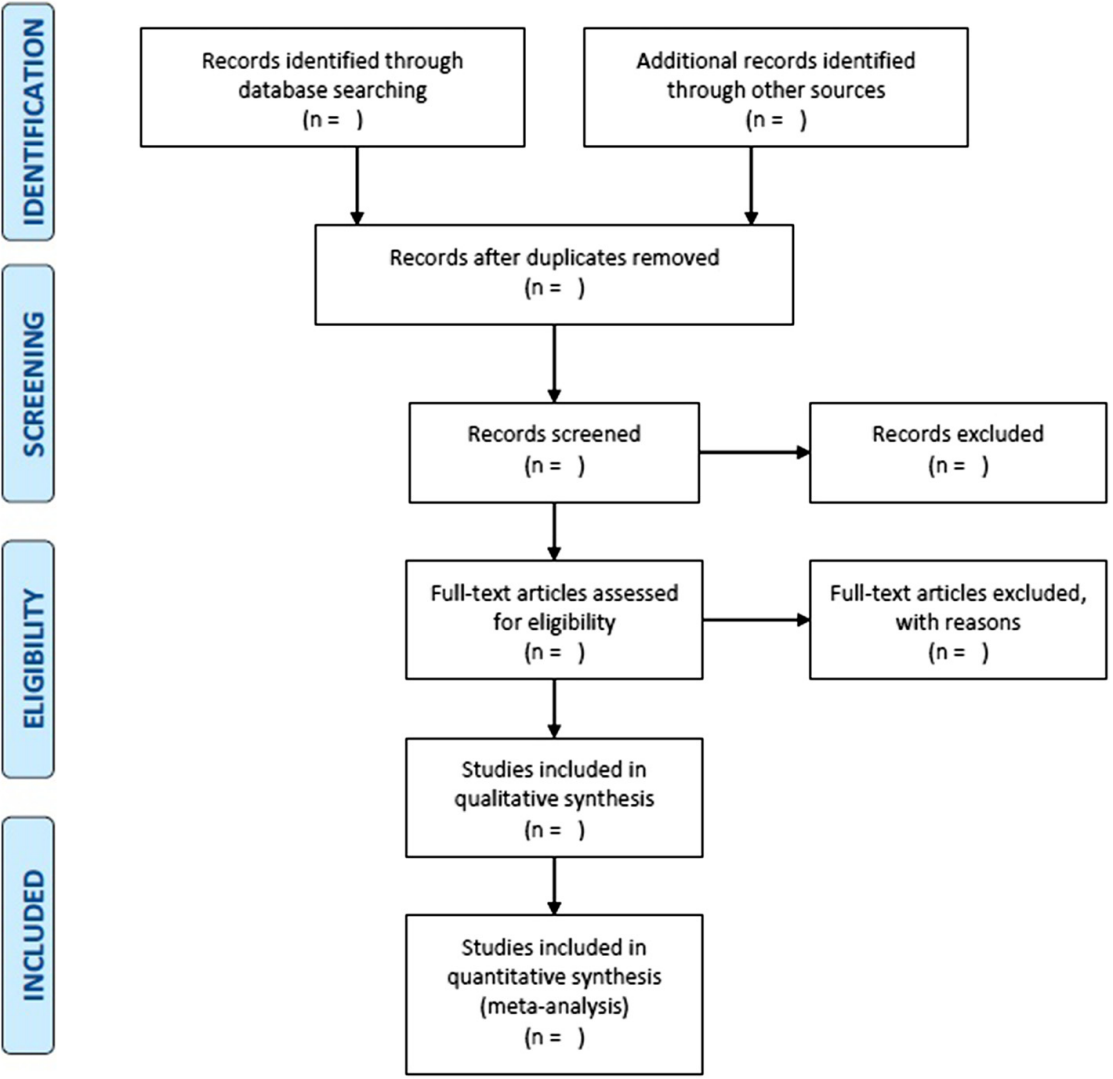
PRISMA flow diagram [14]

We will conduct a systematic review focused on public health and health promotion [12]. The proposed knowledge synthesis approach is the most suitable for the type of interventions under study. Indeed, demand generation interventions in family planning can be considered public health interventions because: (1) they are interconnected with the context in which they are implemented; (2) their conditions of implementation are heterogeneous; and (3) they have an important social dimension, in that they promote sexual health [13]. Given that public health interventions are assessed using a variety of study designs, our systematic review will need to be adjusted to the several types of study designs. Our review will follow the phases of the flow diagram developed by the Preferred Reporting Items for Systematic Reviews and Meta-Analyses (PRISMA) Group (Fig. 1) [14].

### Information sources and search strategy

A search strategy will be developed with a librarian expert at the University of Montreal Hospital Research Centre (CRCHUM) [15]. Bibliographic search filters will be used to identify works published between 1970 and 2015 in English and French. We will begin with the 1970s, as that is when most national voluntary family planning programs were implemented to reduce population growth in developing countries, and in fact, 1970–1990 is considered to be when the “reproductive revolution” occurred in LMICs everywhere, with the exception of sub-Saharan Africa [16].

We will use a combination of medical subject headings (mesh terms) and text words to develop our search strategy. The search strategy will cover four main conceptual categories: (1) low- and middle-income countries, (2) family planning, (3) use and knowledge of contraceptive methods, and (4) interventions. The detailed search strategy is provided in Appendix 1. We will search the following databases: Embase (OVID interface, 1974–2015) CINAHL, Ovid Medline (OVID interface, 1946–2015), Medline (PubMed interface, 1975–2015), and the Cochrane Database of Systematic Reviews. For the gray literature, the following databases will be screened: Google Scholar, Social Care Online, and the National Institute for Health and Care Excellence (NICE). Websites of institutions active in maternal and reproductive health and the bibliographies of potential articles will be used as additional sources of data.

The references will be managed with EndNote software. Two members of the team (BL, AZ) will retrieve the titles and abstracts of articles independently. Any discrepancy will be resolved with the whole team.

### Definition of demand generation interventions

Family planning programs consist of complex interventions that combine enabling environments (policies, strategies, financing, etc.) with demand- and supply-side interventions. In this review, as commissioned by the UNFPA, we will focus on demand generation interventions, which can be defined in several ways. Regardless of how they are defined, demand generation interventions are often heterogeneous and include, but are not limited to the following: “development of advocacy materials for family planning; dissemination of appropriate messages for family planning by community health workers; advocacy on family planning at the community levels to involve the formal and informal leaders; sensitization and awareness creation through community radio, radio drama, television drama, etc.; targeting of special groups including male motivation etc., in the promotion of contraceptives; training of community health/extension workers and others for promotion of family planning; and social marketing of modern contraceptives” [17] and innovative financing for demand generation (such as vouchers and conditional cash transfer).

### Inclusion criteria for studies

In terms of minimum inclusion criteria, we will retain studies that:

1. Looked at family planning interventions and evaluated changes in outcomes that were attributable to the program;
2. Dealt with demand-side activities, even if supply-side activities were also studied; while recognizing that family planning interventions are complex and generally combine both, our focus in this review will be on demand activities, and when both are covered, we will retain only articles in which the results for demand and supply activities are presented separately and will exclude articles in which they are mixed;
3. Were conducted in LMICs, as defined by the World Bank [18];
4. Were related to any or all populations with potential for sexual activity, i.e., women, men, married, unmarried, and adolescents: and
5. In terms of study designs, used randomized controlled trials (RCTs) or cluster randomized trials (CRTs) or were quasi-experimental, e.g., controlled before–after studies (CBAs) and interrupted time series studies (ITSs) will be targeted. The comparator groups for the experimental and quasi-experimental designs to be considered are no intervention or other intervention and pre- and post-tests.

In terms of exclusion criteria, we will not retain studies that:

1. Included only supply-side activities for family planning and contraceptive use;
2. Did not focus on family planning but more largely on youth reproductive health, HIV–AIDS, and STIs;
3. Aimed to assess the feasibility or acceptability of reproductive health programs; and
4. Only described interventions, with no analysis of program impacts.

### Data collection process

We will extract data pertaining to the studies’ characteristics (authors, institutions, country, publication, study design, characteristics of the participants, sample size, description of the interventions) and outcomes/indicators data related to costs, effectiveness, cost-effectiveness, and impacts (Table 1).

**Table 1:** Quantitative data (outcomes) to be extracted from the studies

Category of outcomes	Example of indicator
Costs	
	- Direct costs
	- Indirect costs
	- Health system costs
	- Household costs
	- Household revenue and expenditure
Effectiveness	- Unmet need for modern contraception
	- Use of modern contraceptive method
	- Ever-use of contraception
	- Ever-use of condoms
	- Contraceptive prevalence rate
	- Readiness/intention to use a contraceptive method
	- Changes in knowledge, attitudes, beliefs about FP and/or contraception
	- Discussion of contraception and FP either between partners or between parents and their children
	- Readiness/intention to use a modern method of contraception
Impact	- Fertility
	- Abortion
	- Unintended pregnancies

Two members of the team (BL, AZ) will extract the data independently and compare them. If there is disagreement, a decision will be taken by the whole team.

### Quality assessment

Two members of the research team will also assess the quality of the studies independently. Any discrepancies will be discussed by the whole team, who will resolve them together.

Evaluating the quality of articles documenting public health programs can be challenging due to the variety of study designs used. As such, the evaluation tool used will depend on the study design. For RCTs, we will use the Cochrane Collaboration’s Risk of Bias Tool (CCRBT), and for quasi experimental study designs (CBA, ITS), we will use the criteria of the Cochrane Effective Practice and Organization of Care (EPOC) Group [19]. Thus, the articles will be categorized into three categories of quality: high, medium, and low.

### Data synthesis

If appropriate, a meta-analysis will be conducted using the Cochrane Group’s Review Manager Software (RevMan 5.3) [20]. Analysis will involve comparing odd ratio (OR) Outcomes will be compared between control and intervention groups. For dichotomous data, we will use ORs with 95 % confidence intervals (CI) as measures of effect. Standardized mean difference (SMD) will be used for continuous data using 95 % CIs as measures of effect size.

To evaluate study designs such as interrupted time series, we will use auto regressive integrated moving average (ARIMA) models, including them in the meta-analysis [21]. Because the validity of a meta-analysis depends on the exploration of the heterogeneity of results, we will pay particular attention to this element. We will use the *I*^2^ index and *Q* statistic to assess heterogeneity, adopting a 50 % standard as recommended by Cochrane guidelines [19].

In the meta-analysis, two statistical procedures could be used: *fixed* or *random effects*. Fixed effects model assumes any difference in effect size in the meta-analysis is due only to sampling error. In that model, all studies share a common mean. In contrast, random effects model assumes variation not only in the sampling, but also in how studies are conducted [22]. Because we expect considerable variation in the studies retained, we will use random effects to gain a better understanding of the results.

If heterogeneity exceeds 50 %, we will undertake subgroup analyses and meta-regression. For example, we might conduct subgroup analyses related to study designs, year of publication, settings, and types of intervention. However, if we find too much heterogeneity in outcomes measures, as has been found in several systematic reviews, we will present the results narratively [5, 23–26].

The report will be presented in a format geared toward knowledge users [27] as well as to the PRISMA statement and checklist [14]. This protocol is registered in PROSPERO (CRD 42015017549).

## Discussion

We know what works to improve outcomes related to family planning programs. However, we do not yet know how to do it effectively [28, 29]. By increasing knowledge on the cost-effectiveness of demand generation interventions, this systematic review will provide evidence to support the improvement of family planning programs. It will also identify knowledge gaps regarding family planning interventions.

Recommendations will be made to the UNFPA and partner agencies in the Family Planning 2020 community. This review will also contribute to the development of a guidance document and programmatic tools for planning, implementing, and evaluating demand generation interventions in family planning as part of the UNFPA Global Programme to Enhance Reproductive Health Commodity Security (GPRHCS). The program aims to “ensure a secure, steady and reliable supply of quality reproductive health commodities and improve access and use” by strengthening national health systems and services [3, p. 9].

The evidence gathered from this review will also be used to enhance the effectiveness of family planning programs in LMICs. Family planning is considered to be a primary prevention measure and an efficient public health intervention [28]. Indeed, family planning is closely entwined with maternal health. The use of modern contraceptive methods could prevent up to one third of maternal deaths. Effective family planning programs are a critical means of empowering women and adolescent girls, improving human capital, reducing dependency ratios, reducing maternal (through mortality depletion) and child (through birth spacing and improved nutrition) mortality, and achieving demographic dividends in low- and middle-income countries [28].

Nevertheless, family planning remains underused and still often neglected by some governments [28, 29]. No country in the world has achieved sustainable development without meeting a high percentage of the demand for modern contraception [30]. Indeed, in a recent working paper, Kendall [31] noted that family planning in LMICs has one of the greatest coverage gaps globally. Since 2000, the demand for family planning has risen very slowly [32]. In WHO’s Countdown to 2015 report, the median coverage for the 37 countries with data is 61 %, with a range of 15 % (Chad) to 95 % (Vietnam) [33]. Kendall [31] pointed to family planning as an area of critical maternal health knowledge gaps in LMICs for post-2015. As such, increasing knowledge on family planning remains a global health priority for the agenda beyond 2015. Lastly, our results will be published in policy briefs as well as in a peer-reviewed scientific journal.

## Appendix 1

### Search strategy on effect of demand generation interventions on uptake and use of modern contraceptives in LMIC

Group 1 (concept: low- and middle-income)

“Afghanistan”[Mesh] OR “Libya”[Mesh] OR “Albania”[Mesh] OR “Macedonia Republic”[Mesh] OR “Algeria”[Mesh] OR “Madagascar”[Mesh] OR “American Samoa”[Mesh] OR “Malawi”[Mesh] OR “Angola”[Mesh] OR “Malaysia”[Mesh] OR “Argentina”[Mesh] OR “Indian Ocean Islands”[Mesh] OR “Armenia”[Mesh] OR “Mali”[Mesh] OR “Azerbaijan”[Mesh] OR “Micronesia”[Mesh] OR “Bangladesh”[Mesh] OR “Mauritania”[Mesh] OR “Republic of Belarus”[Mesh] OR “Mauritius”[Mesh] OR “Belize”[Mesh] OR “Mexico”[Mesh] OR “Benin”[Mesh] OR “Bhutan”[Mesh] OR “Moldova”[Mesh] OR “Bolivia”[Mesh] OR “Mongolia”[Mesh] OR “Bosnia-Herzegovina”[Mesh] OR “Montenegro”[Mesh] OR “Botswana”[Mesh] OR “Morocco”[Mesh] OR “Brazil”[Mesh] OR “Mozambique”[Mesh] OR “Bulgaria”[Mesh] OR “Myanmar”[Mesh] OR “Burkina Faso”[Mesh] OR “Namibia”[Mesh] OR “Burundi”[Mesh] OR “Nepal”[Mesh] OR “Nicaragua”[Mesh] OR “Cambodia”[Mesh] OR “Niger”[Mesh] OR “Cameroon”[Mesh] OR “Nigeria”[Mesh] OR “Central African Republic”[Mesh] OR “Pakistan”[Mesh] OR “Chad”[Mesh] OR “Palau”[Mesh] OR “China”[Mesh] OR “Panama”[Mesh] OR “Colombia”[Mesh] OR “Papua New Guinea”[Mesh] OR “Comoros”[Mesh] OR “Paraguay”[Mesh] OR “Democratic Republic of the Congo”[Mesh] OR “Congo”[Mesh] OR “Peru”[Mesh] OR “Philippines”[Mesh] OR “Costa Rica”[Mesh] OR “Romania”[Mesh] OR “Cote d'Ivoire“[Mesh] OR “Rwanda”[Mesh] OR “Cuba”[Mesh] OR “Samoa”[Mesh] OR “Djibouti”[Mesh] OR “Atlantic Islands”[Mesh] OR “Dominica”[Mesh] OR “Senegal”[Mesh] OR “Dominican Republic”[Mesh] OR “Serbia”[Mesh] OR “Ecuador”[Mesh] OR “Seychelles”[Mesh] OR “Egypt”[Mesh] OR “Sierra Leone”[Mesh] OR “El Salvador”[Mesh] OR “Melanesia”[Mesh] OR “Eritrea”[Mesh] OR “Somalia”[Mesh] OR “Ethiopia”[Mesh] OR “South Africa”[Mesh] OR “Fiji”[Mesh] OR “Sudan”[Mesh] OR “Gabon”[Mesh] OR “Sri Lanka”[Mesh] OR “Gambia”[Mesh] OR “Saint Lucia”[Mesh] OR “Georgia”[Mesh] OR “Saint Vincent and the Grenadines”[Mesh] OR “Ghana”[Mesh] OR “Grenada”[Mesh] OR “Suriname”[Mesh] OR “Guatemala”[Mesh] OR “Swaziland”[Mesh] OR “Guinea”[Mesh] OR “Syria”[Mesh] OR “Guinea-Bissau”[Mesh] OR “Tajikistan”[Mesh] OR “Guyana”[Mesh] OR “Tanzania”[Mesh] OR “Haiti”[Mesh] OR “Thailand”[Mesh] OR “Honduras”[Mesh] OR “East Timor”[Mesh] OR “Hungary”[Mesh] OR “Togo”[Mesh] OR “India”[Mesh] OR “Tonga”[Mesh] OR “Indonesia”[Mesh] OR “Tunisia”[Mesh] OR “Iran”[Mesh] OR “Turkey”[Mesh] OR “Iraq”[Mesh] OR “Turkmenistan”[Mesh] OR “Jamaica”[Mesh] OR “Micronesia”[Mesh] OR “Jordan”[Mesh] OR “Uganda”[Mesh] OR “Kazakhstan”[Mesh] OR “Ukraine”[Mesh] OR “Kenya”[Mesh] OR “Uzbekistan”[Mesh] OR “Vanuatu”[Mesh] OR “Korea”[Mesh] OR “Venezuela”[Mesh] OR “Kosovo”[Mesh] OR “Vietnam”[Mesh] OR “Kyrgyzstan”[Mesh] OR “Middle East”[Mesh] OR “Yemen”[Mesh] OR “Lebanon”[Mesh] OR “Lesotho”[Mesh] OR “Zambia”[Mesh] OR “Zimbabwe”[Mesh] OR “Liberia”[Mesh] OR “Developing Countries”[Mesh] OR Afghanistan[Title/Abstract] OR Albania[Title/Abstract] OR Algeria[Title/Abstract] OR American Samoa[Title/Abstract] OR Angola[Title/Abstract] OR Argentina[Title/Abstract] OR Armenia[Title/Abstract] OR Azerbaijan[Title/Abstract] OR Bangladesh[Title/Abstract] OR Belarus[Title/Abstract] OR Belize[Title/Abstract] OR Benin[Title/Abstract] OR Bhutan[Title/Abstract] OR Bolivia[Title/Abstract] OR Bosnia Herzegovina[Title/Abstract] OR Botswana[Title/Abstract] OR Brazil[Title/Abstract] OR Bulgaria[Title/Abstract] OR Burkina Faso[Title/Abstract] OR Burundi[Title/Abstract] OR Cabo Verde[Title/Abstract] OR Cambodia[Title/Abstract] OR Cameroon[Title/Abstract] OR Central African Republic[Title/Abstract] OR Chad[Title/Abstract] OR China[Title/Abstract] OR Colombia[Title/Abstract] OR Comoros[Title/Abstract] OR Congo[Title/Abstract] OR Costa Rica[Title/Abstract] OR Cote d'Ivoire[Title/Abstract] OR Cuba[Title/Abstract] OR Djibouti[Title/Abstract] OR Dominica[Title/Abstract] OR Dominican Republic[Title/Abstract] OR Ecuador[Title/Abstract] OR Egypt[Title/Abstract] OR El Salvador[Title/Abstract] OR Eritrea[Title/Abstract] OR Ethiopia[Title/Abstract] OR Fiji[Title/Abstract] OR Gabon[Title/Abstract] OR Gambia[Title/Abstract] OR Georgia[Title/Abstract] OR Ghana[Title/Abstract] OR Grenada[Title/Abstract] OR Guatemala[Title/Abstract] OR Guinea[Title/Abstract] OR Guinea-Bissau[Title/Abstract] OR Guyana[Title/Abstract] OR Haiti[Title/Abstract] OR Honduras[Title/Abstract] OR Hungary[Title/Abstract] OR India[Title/Abstract] OR Indonesia[Title/Abstract] OR Iran[Title/Abstract] OR Iraq[Title/Abstract] OR Jamaica[Title/Abstract] OR Jordan[Title/Abstract] OR Kazakhstan[Title/Abstract] OR Kenya[Title/Abstract] OR Kiribati[Title/Abstract] OR Korea[Title/Abstract] OR Kosovo[Title/Abstract] OR Kyrgyz [Title/Abstract] OR Lao PDR[Title/Abstract] OR Lebanon[Title/Abstract] OR Lesotho[Title/Abstract] OR Liberia[Title/Abstract] OR Libya[Title/Abstract] OR Macedonia[Title/Abstract] OR Madagascar[Title/Abstract] OR Malawi[Title/Abstract] OR Malaysia[Title/Abstract] OR Maldives[Title/Abstract] OR Mali[Title/Abstract] OR Marshall Islands[Title/Abstract] OR Mauritania[Title/Abstract] OR Mauritius[Title/Abstract] OR Mexico[Title/Abstract] OR Micronesia[Title/Abstract] OR Moldova[Title/Abstract] OR Mongolia[Title/Abstract] OR Montenegro[Title/Abstract] OR Morocco[Title/Abstract] OR Mozambique[Title/Abstract] OR Myanmar[Title/Abstract] OR Namibia[Title/Abstract] OR Nepal[Title/Abstract] OR Nicaragua[Title/Abstract] OR Niger[Title/Abstract] OR Nigeria[Title/Abstract] OR Pakistan[Title/Abstract] OR Palau[Title/Abstract] OR Panama[Title/Abstract] OR Papua New Guinea[Title/Abstract] OR Paraguay[Title/Abstract] OR Peru[Title/Abstract] OR Philippines[Title/Abstract] OR Romania[Title/Abstract] OR Rwanda[Title/Abstract] OR Samoa[Title/Abstract] OR Sao Tome[Title/Abstract] OR Senegal[Title/Abstract] OR Serbia[Title/Abstract] OR Seychelles[Title/Abstract] OR Sierra Leone[Title/Abstract] OR Solomon Islands[Title/Abstract] OR Somalia[Title/Abstract] OR South Africa[Title/Abstract] OR South Sudan[Title/Abstract] OR Sri Lanka[Title/Abstract] OR St. Lucia[Title/Abstract] OR St. Vincent the Grenadines[Title/Abstract] OR Sudan[Title/Abstract] OR Suriname[Title/Abstract] OR Swaziland[Title/Abstract] OR Syrian Arab Republic[Title/Abstract] OR Tajikistan[Title/Abstract] OR Tanzania[Title/Abstract] OR Thailand[Title/Abstract] OR Timor-Leste[Title/Abstract] OR Togo[Title/Abstract] OR Tonga[Title/Abstract] OR Tunisia[Title/Abstract] OR Turkey[Title/Abstract] OR Turkmenistan[Title/Abstract] OR Tuvalu[Title/Abstract] OR Uganda[Title/Abstract] OR Ukraine[Title/Abstract] OR Uzbekistan[Title/Abstract] OR Vanuatu[Title/Abstract] OR Venezuela[Title/Abstract] OR Vietnam[Title/Abstract] OR Gaza[Title/Abstract] OR Yemen[Title/Abstract] OR Zambia[Title/Abstract] OR Zimbabwe[Title/Abstract] OR “Developing Countr*”[Title/Abstract] or middle income countr* or low income countr*).

AND

Groupe 2 (concept: family planning)

- birth control[Title/Abstract] OR “Family Planning Services”[Mesh:NoExp] OR “Family Planning Policy”[Mesh:NoExp] OR “Contraception Behavior”[Mesh:NoExp] OR “Contraception/psychology[Mesh:NoExp] OR “Contraception/utilization”[Mesh:NoExp] OR family planning[Title/Abstract] OR contracepti*[Title/Abstract] OR planned pregnanc*[Title/Abstract] OR birth prevention[Title/Abstract] OR prevent* pregnanc*[Title/Abstract] OR “Birth Intervals” [Mesh:NoExp] OR birth interval*[Title/Abstract] OR birth spacing[Title/Abstract] OR pregnancy interval[Title/Abstract] OR pregnancy spacing[Title/Abstract]

AND

Group 3 (concept: use and knowledge of contraceptive methods)

- Health knowledge

Contraceptive use

Attitudes, practice

Health promotion/methods

Health services needs and demand

Costs and cost analysis

Cost–benefit analysis

Health impact assessment

Program evaluation

Demand generation, awareness

AND

Group 4 (concept: intervention)

- Quasi experimental studies
- Randomized controlled trial
- Pre–post test
- Intervention studies

## Abbreviations

AIDS: acquired immunodeficiency syndrome
ARIMA: auto regressive regressive integrated moving average
CBA: controlled before–after studies
CCRBT: Cochrane Collaboration’s Risk of Bias Tool
CI: confidential intervals
CRCHUM: University of Montreal Hospital Research Centre
CRCT: cluster randomized controlled trials
GPRHCS: Global Programme Reproductive Health Commodity Security
HIV: human immunodeficiency virus
ITS: interrupted time series studies
LMIC: low–middle-income countries
NICE: National Institute for Health and Care Excellence
RCT: randomized controlled trials
RR: relative risk
SMD: standardized mean difference
STI: sexually transmitted infections
UNFPA: United Nations Population Fund
WHO: World Health Organization
